# Inhibition of oxygen-sensing prolyl hydroxylases increases lipid accumulation in human primary tubular epithelial cells without inducing ER stress

**DOI:** 10.1007/s00441-020-03186-w

**Published:** 2020-03-18

**Authors:** Gunnar Schley, Steffen Grampp, Margarete Goppelt-Struebe

**Affiliations:** Department of Nephrology and Hypertension, Friedrich-Alexander University Erlangen-Nürnberg and University Hospital Erlangen, Loschgestrasse 8, 91054 Erlangen, Germany

**Keywords:** Prolyl hydroxylase inhibitors, Cyclosporine A, Human primary tubular epithelial cells, ER stress, Lipid accumulation

## Abstract

The role of the hypoxia-inducible transcription factor (HIF) pathway in renal lipid metabolism is largely unknown. As HIF stabilizing prolyl hydroxylase (PHD) inhibitors are currently investigated in clinical trials for the treatment of renal anemia, we studied the effects of genetic deletion and pharmacological inhibition of PHDs on renal lipid metabolism in transgenic mice and human primary tubular epithelial cells (hPTEC). Tubular cell-specific deletion of HIF prolyl hydroxylase 2 *(Phd2)* increased the size of Oil Red-stained lipid droplets in mice. In hPTEC, the PHD inhibitors (PHDi) DMOG and ICA augmented lipid accumulation, which was visualized by Oil Red staining and assessed by microscopy and an infrared imaging system. PHDi-induced lipid accumulation required the exogenous availability of fatty acids and was observed in both proximal and distal hPTEC. PHDi treatment was not associated with structural features of cytotoxicity in contrast to treatment with the immunosuppressant cyclosporine A (CsA). PHDi and CsA differentially upregulated the expression of the lipid droplet-associated genes *PLIN2*, *PLIN4* and *HILPDA*. Both PHDi and CsA activated AMP-activated protein kinase (AMPK) indicating the initiation of a metabolic stress response. However, only CsA triggered endoplasmic reticulum (ER) stress as determined by the increased mRNA expression of multiple ER stress markers but CsA-induced ER stress was not linked to lipid accumulation. Our data raise the possibility that PHD inhibition may protect tubular cells from toxic free fatty acids by trapping them as triacylglycerides in lipid droplets. This mechanism might contribute to the renoprotective effects of PHDi in experimental kidney diseases.

**Electronic supplementary material:**

The online version of this article (10.1007/s00441-020-03186-w) contains supplementary material, which is available to authorized users.

## Introduction

Accumulation of excess lipids in non-adipose tissues is associated with cellular dysfunction and injury (Weinberg 2006). So-called lipotoxicity has been extensively studied in skeletal and cardiac myocytes, hepatocytes and pancreatic β-cells, particularly in the context of metabolic syndrome. Now lipotoxicity is also recognized to contribute to the development of acute kidney injury (AKI) and the progression of chronic kidney disease (CKD) (Izquierdo-Lahuerta et al. 2016). Toxicity is mainly derived from nonesterified fatty acids and their metabolites (Weinberg 2006).

Fatty acids (FA) are the best energy-yielding substrates producing three times more ATP than glucose. They are the preferred substrates of proximal tubular cells (Guder et al. 1986; Silva 1990), which have a high energy demand for the reabsorption of solutes, mainly sodium, from the glomerular filtrate (Layton et al. 2016). FA are mostly bound to albumin and enter the proximal tubular cell from the basolateral surface in linear correlation to their arterial concentration (Wirthensohn and Guder 1986). In the presence of high urinary amounts of albumin in proteinuric kidney diseases, FA can also be obtained from the glomerular filtrate across the luminal surface (Bobulescu 2010; Moorhead et al. 1982). De novo synthesis of FA is insignificant in the kidneys (Wirthensohn and Guder 1983). FA can either be degraded by β-oxidation or can be incorporated into triacylglycerols and phospholipids. Proximal tubular cells contain the whole enzymatic machinery for mitochondrial and peroxisomal FA oxidation as well as for triacylglycerol synthesis (Guder and Ross 1984). Depending on the nutritional state, triacylglycerols are stored in lipid droplets in proximal tubular cells (Wirthensohn and Guder 1986). Renal lipid accumulation was observed during fasting (Scerbo et al. 2017; Wirthensohn and Guder 1986) and in kidney disorders related to aging (Jiang et al. 2005a), obesity (Futatsugi et al. 2016; Jiang et al. 2005b), diabetes (Sun et al. 2002) and AKI following various causes (Johnson et al. 2005; Portilla et al. 2006; Tran et al. 2016; Zager et al. 2005). Potential mechanisms leading to tubular lipid accumulation include increased FA uptake and synthesis as well as diminished β-oxidation of FA but the relative importance of each mechanism is still unclear. In human kidney biopsies with CKD and in mouse models of kidney fibrosis, enzymes and regulators of FA oxidation were found to be downregulated (Kang et al. 2015). However, in proximal tubular epithelial cells isolated from proteinuric human patients and in differentiated proximal tubules isolated from fibrotic mouse kidneys, the FA oxidation was instead upregulated (Bataille et al. 2018; Rudnicki et al. 2007).

Hypoxia has a crucial role in the pathophysiology of both AKI and CKD (Nangaku et al. 2013). Cellular adaptive responses to hypoxia are mediated by hypoxia-inducible transcription factors (HIF). The oxygen-dependent degradation of the HIFα subunit under normoxic conditions involves prolyl hydroxylases (PHDs) and the von Hippel-Lindau (VHL) protein (Schodel and Ratcliffe 2019). In contrast to the well-established role of HIF in glucose metabolism, its function in lipid metabolism is poorly characterized. Under hypoxic conditions, HIF promotes cellular FA uptake, synthesis and storage and suppresses FA oxidation in cancer cells (Samanta and Semenza 2018)*.* In several tumor cell lines, various lipid metabolic genes were identified as direct HIF transcriptional targets (Mylonis et al. 2019). A *Vhl*-deficient mouse model resembling characteristics of human clear cell renal cell carcinoma (ccRCC) exhibited lipid accumulation in proximal tubules (Fu et al. 2011). However, apart from ccRCC, the role of HIF in renal lipid metabolism has not been determined.

In this study, we investigate whether HIF stabilization in vivo and in vitro results in lipid accumulation in renal tubular cells. In mice with tubular cell-specific deletion of *Phd2*, lipid droplets accumulated in proximal tubular cells. HIF stabilizing PHD inhibitors (PHDi) are currently evaluated in phase 3 clinical trials for the treatment of renal anemia (Maxwell and Eckardt 2016; Sugahara et al. 2017). In human primary tubular epithelial cells (hPTEC) of proximal and distal tubular origin, PHDi increased lipid storage. In contrast to the immunosuppressant cyclosporine A, which is known to promote lipotoxicity (Lhotak et al. 2012; Mihatsch et al. 1988), PHDi-induced lipid accumulation was not associated with cell injury or endoplasmic reticulum (ER) stress. Our results suggest that storage of exogenous fatty acids in lipid droplets might contribute to the renoprotective effects of PHDi in experimental kidney diseases (Schley et al. 2019; Schley et al. 2012).

## Material and methods

### Materials

AEBSF (4-(2-Aminoethyl) benzenesulfonyl fluoride hydrochloride), cyclosporine A and DMOG (dimethyloxalyl glycine) were obtained from Cayman Chemicals. ICA (2-(1-chloro-4-hydroxyisoquinoline-3-carboxamido) acetate) was synthesized, as previously described (Schley et al. 2012). Stabilization of HIFα and upregulation of HIF target gene expression by DMOG and ICA have been shown earlier (Kroening et al. 2010; Schley et al. 2012). For cell culture experiments, compounds were dissolved in dimethyl sulfoxide (DMSO), which was used as vehicle control. The final concentration of DMSO did not exceed 0.1%, which did not affect the parameters measured.

### Cell culture

Human primary tubular epithelial cells (hPTEC) were isolated from renal cortical tissues collected from healthy parts of tumor nephrectomies, as previously described (Grampp et al. 2016; Keller et al. 2012; Kroening et al. 2010; Muller et al. 2018). Isolation of human cells was approved by the local ethics committee (Reference number 3755, Ethik-Kommission der Medizinischen Fakultät der FAU Erlangen-Nürnberg). Written informed consent was obtained from all donors. In brief, renal cortical tissue was minced on ice, digested by DNAse I (Roche Diagnostics) and collagenase II (Gibco). Tubular cells were obtained by sieving through 100- and 70-μm disposable filters. Cells were seeded in epithelial cell-selective medium (DMEM/Ham’s F12 medium containing 2 mM L-glutamine, 100 U/ml penicillin, 100 μg/ml streptomycin, insulin-transferrin-selenium supplement, 10 ng/ml epidermal growth factor, 36 ng/ml hydrocortisone and 4 pg/ml triiodothyronine) supplemented with 10% fetal calf serum (FCS) for 1–2 days. Debris was removed and the cells were further cultured in FCS-free medium. After about 1 week, a culture of tubular epithelial cells was obtained that stained positive for keratins (Muller et al. 2018) and either E- or N-cadherin (Keller et al. 2012; Kroening et al. 2010). A detailed cell isolation protocol is available upon request from the authors.

For experiments, hPTEC were seeded in epithelial cell-selective medium containing 2.5% FCS to facilitate cell attachment. After 24 h, cells were switched to FCS-free medium. hPTEC at passages 1–3 were used for the experiments. Cells of proximal and distal tubular origin were separated by their differential adherence to cell culture plastic material with distal cells being more adhesive than proximal hPTEC. Cell trypsinization for 3 min resulted in a culture enriched for proximal tubular cells (about 60% N-cadherin positive cells) and a fraction of E-cadherin positive cells. Additional trypsinization was necessary to remove the remaining cells from the cell culture dish. These cells were more than 90% positive for E-cadherin and thus represented cells of distal tubular origin (Keller et al. 2012; Kroening et al. 2010; Preisser et al. 2016).

### Quantification of cell numbers and Oil Red intensity in 48-well plates

hPTEC (40,000 cells per well of a 48-well plate) were seeded in quadruplicates in epithelial cell-selective medium containing 0.5% FCS. After 24 h, cells were further incubated in medium without FCS in the presence of 0.5% bovine serum albumin (BSA) (PAA Laboratories) or BSA essentially fatty acid free (#6003, Sigma-Aldrich). To avoid variations in the lipid content, one batch of BSA was used throughout the whole study. At the end of the incubation period, cells were fixed with 3.5% paraformaldehyde (PFA) for 10 min and washed twice with water for 5 min and with 60% isopropanol for 5 min.

Oil Red (OR) staining was performed following established protocols (Kinkel et al. 2004; Mehlem et al. 2013). Prior to use, OR stock solution (5 mg/ml in 60% triethyl phosphate, Sigma-Aldrich) was diluted to 3 mg/ml with ddH_2_O yielding OR working solution. Cells were stained with OR working solution for 20 min in the dark and washed with ddH_2_O for three times. Nuclei were stained with DAPI (4′,6-diamidine-2′-phenylindole dihydrochloride, Sigma-Aldrich) and analyzed within 24 h. OR staining intensity was quantified simultaneously in all wells of one 48-well plate using the Odyssey infrared imaging system (LI-COR, Biosciences). DAPI staining was imaged using a Keyence BZ-9000 inverted microscope. Cell numbers were determined by ImageJ software version 1.51 (Schneider et al. 2012). For each well, OR staining intensity was normalized to the cell number. The mean value of control cells was set to 1 in each experiment.

### Quantification of Oil Red intensity by immunocytochemistry

hPTEC (50,0000 cells per well) were seeded on collagen IV-coated coverslips placed in 24-well plates and incubated as indicated in the figure legends. Cells were fixed and stained with OR working solution as described above. Subsequently, cells were incubated with blocking solution (1% BSA in phosphate-buffered saline (PBS)) for 1 h at room temperature and then with a mouse monoclonal anti-E-cadherin antibody (ab1416, Abcam, 1:200) overnight. PromoFluor® anti-mouse antibody was used as secondary antibody (Promokine, 1:500). Cell nuclei were stained with DAPI. Thereafter, cells were mounted in Mowiol. To visualize lipid droplets in different layers of the cells, image stacks were acquired with a Keyence BZ-9000 fluorescence microscope and automatically composed to a fully-focused image using BZ-II analyzer software (Keyence). Photos of E-cadherin and DAPI stainings were taken in parallel. For quantification purposes, areas were chosen that contained colonies of proximal and distal tubular cells, respectively, on one slide. For each image, cell number and OR staining intensity were determined separately for proximal and distal tubular cells using ImageJ software. Data are presented as ratio of distal to proximal intensity per cell determined on the same slide.

### Western blot analysis

For Western blot analysis, hPTEC (250,000 cells per well) were seeded in 12-well plates and incubated as described above. Cell homogenates were collected in a modified RIPA buffer containing 50 mM HEPES pH 7.4, 150 mM NaCl, 1% Triton X-100, 1 mM EDTA, 10% glycerol, 2 mM sodium vanadate and EDTA-free protease inhibitor cocktail (cOmplete™, Roche Diagnostics) or in PBS containing 5% SDS and protease inhibitors to detect phosphorylated proteins. Western blot analyses were performed essentially as described before (Kroening et al. 2009) using the following antibodies: rabbit monoclonal anti-AMPKα (D63G4) #5832 and rabbit monoclonal anti-phospho-AMPKα (Thr172) (40H9) #2535 from Cell Signaling Technology, rabbit polyclonal anti-GRP78 antibody (ab21685) from Abcam, rabbit polyclonal anti-vinculin (H-300) (sc-5573) from Santa Cruz and donkey anti-rabbit IgG (NA934V) from Amersham Biosciences. The immunoreactive bands were quantified using the Luminescent Image Analyzer LAS-1000 (Fujifilm) and AIDA 4.15 Image Analyzer software (Raytest). To compare blots of different experiments, band intensities were normalized to those of control cells on each blot.

### RNA preparation and real-time PCR

Total RNA was extracted from hPTEC using the peqGOLD Total RNA Kit (VWR Peqlab) and reverse transcribed into cDNA using the High-Capacity cDNA Reverse Transcription Kit (Thermo Fisher Scientific). Quantitative real-time PCR (RT-PCR) was performed with Fast SYBR™ Green (Applied Biosystems) on a StepOnePlus™ Real-Time PCR system (Applied Biosystems). Primer sequences are listed in Electronic Supplementary Material, Table S1. Primer sequences used for the detection of *ATF6*, *CHOP*, *ECAD*, *GRP78*, *GRP94*, *HERPNCAD* and *sXBP1* have been previously described (Bouvier et al. 2009; Bouvier et al. 2012; Fougeray et al. 2011; Keller et al. 2012). Gene expression was normalized to *HPRT* and relative fold changes in gene expression were calculated using the comparative 2^−ΔΔCt^ method.

### Animal experiments

All animal experiments were approved by the animal care and use committee of local government authorities (Regierung von Mittelfranken, Ansbach, Germany; Az 54-2532.1-11/13) and conducted in accordance with the Guide for the Care and Use of Laboratory Animals (National Research Council 2011). Mice with renal tubular cell-specific knockout of *Phd2* (*Phd2*^ΔKsp^) were obtained by crossing C57BL/6 mice carrying loxP-flanked *Phd2* alleles to C57BL/6 mice harboring Cre recombinase under control of the kidney-specific cadherin (Ksp1.3) promoter, as described earlier (Schley et al. 2015). Cre-negative littermates were used as wild-type controls. Generation and genotyping of Ksp1.3-Cre and loxP-Phd2 mice have been described elsewhere (Shao et al. 2002; Singh et al. 2013). The animals were housed under standard conditions (room temperature 22 ± 1 °C, humidity 55 ± 5%, 12:12 h light-dark cycle) with free access to standard rodent chow (V1534-000, ssniff Spezialdiäten) and tap water ad libitum. Twenty-week-old male mice were sacrificed by exsanguination under deep isoflurane anesthesia. Kidneys were either embedded in Tissue-Tek® O.C.T.™ compound (Sakura Finetek) and snap frozen in liquid nitrogen or fixed by transcardial perfusion with 4% PFA.

Frozen kidney sections (3 μm) were stained for 5 min with OR working solution in the dark. The size of lipid droplets was determined in 6 areas of the renal cortex from 3 mice in each group at 200-fold magnification using ImageJ software version 1.51. For immunohistochemical detection of sodium phosphate cotransporter (NaPi) IIa, frozen kidney sections were incubated with the following antibodies: rabbit polyclonal anti-rat NaPi-IIa (Custer et al. 1994) (diluted 1:150 in Dako Antibody Diluent) overnight at 4 °C followed by FITC-conjugated goat polyclonal anti-rabbit antibody (Vector Laboratories, FI-1000; diluted 1:500 in PBS with 1% BSA) for 30 min at room temperature.

PFA-fixed and paraffin-embedded kidney sections (2 μm) were stained with Periodic acid-Schiff (PAS) reagent. Microphotographs were acquired using a DMR microscope equipped with a DMC6200 camera from Leica Microsystems or an Eclipse 80i microscope with a DS-Qi2 camera from Nikon Instruments.

### Statistical analysis

If not indicated otherwise, numbers of experiments refer to isolations of cells from different patients. Two groups were compared with Student’s *t* test. Multiple samples were compared by ANOVA with an appropriate post hoc test using GraphPad Prism version 5.04 for Windows (GraphPad Software). A *p* value of *p* < 0.05 was considered as indication of statistical significance.

## Results

### Loss of tubular Phd2 leads to lipid accumulation in mice

**Fig. 1:**
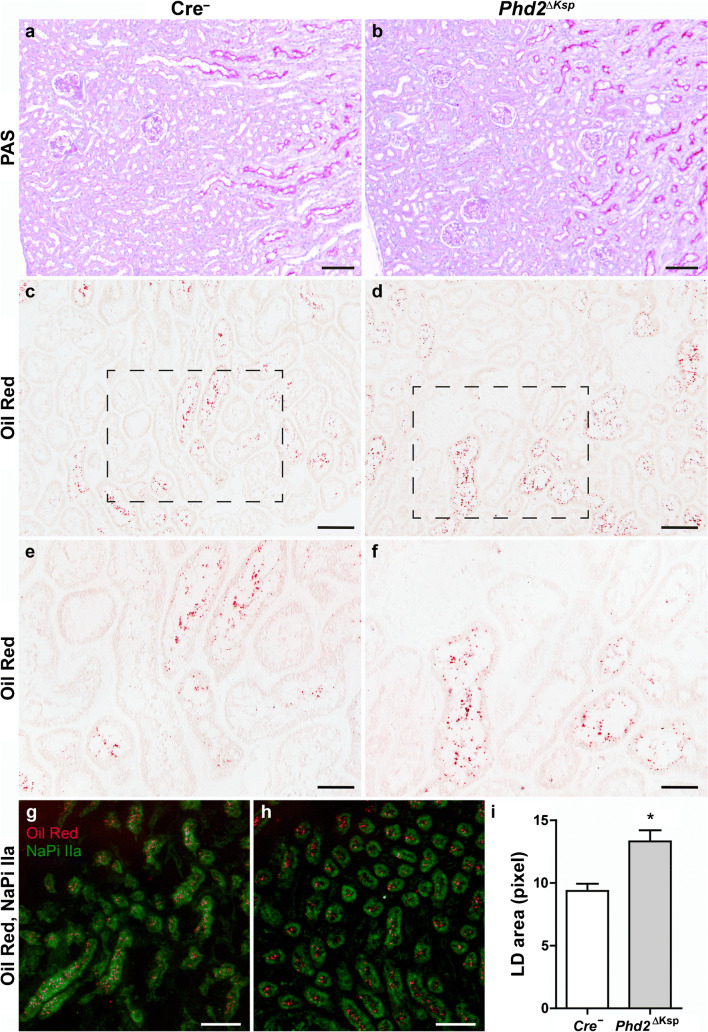
Increased lipid accumulation in tubulular cells of *Phd2* knockout mice. Kidney sections from mice with renal tubular-specific deficiency of *Phd2* (*Phd2*^*ΔKsp*^) and their Cre^−^ littermates were stained with PAS (**a**, **b**), Oil Red (**c**–**h**) and for sodium phosphate cotransporter (NaPi) IIa (**g**, **h**). **e**, **f** Higher magnification of the renal cortical region in the dotted box from (**c**) and (**d**), respectively. **g**, **h** Staining of Oil Red (red) and NaPi IIa (green) was merged. Scale bars: (**a**, **b**) 100 μm, (**c**, **d**, **g**, **h**) 50 μm, (**e**, **f**) 25 μm. **i** The area of Oil Red-stained lipid droplets (LD) was quantified in the renal cortex of *Phd2*^*ΔKsp*^ (*n* = 3) and Cre^−^ mice (*n* = 3) by computer image analysis. Data are means ± SEM, *p < 0.05, two-sided *t* test

To test our hypothesis that HIF stabilization increases lipid accumulation in the kidneys, we compared mice with Ksp1.3-Cre directed tubular cell-specific deletion of *Phd2* (*Phd2*^ΔKsp^) and their Cre^−^ littermates (Schley et al. 2015). PHD2 is the dominant PHD isoform in renal tubular cells (Schodel et al. 2009). PAS stainings of wild-type and *Phd2*^ΔKsp^ kidneys revealed no apparent tubular or interstitial pathology (Fig. 1a, b). Using Oil Red (OR) staining, lipid droplets were detected in individual tubular cells scattered throughout the renal cortex of both Cre^−^ and *Phd2*^ΔKsp^ mice (Fig. 1c–f), which were identified as proximal tubular cells by co-immunostaining for the sodium phosphate cotransporter (NaPi) IIa (Fig. 1g, h). In *Phd2*^ΔKsp^ mice, the average size of lipid droplets was significantly increased (Fig. 1i). Ninety percent of lipid droplets in *Phd2*^ΔKsp^ mice were larger than the average lipid droplet in Cre^−^ littermates. This observation prompted us to further investigate the role of PHDs for lipid accumulation in cultured tubular epithelial cells.

### Characterization of human primary tubular epithelial cells

**Fig. 2:**
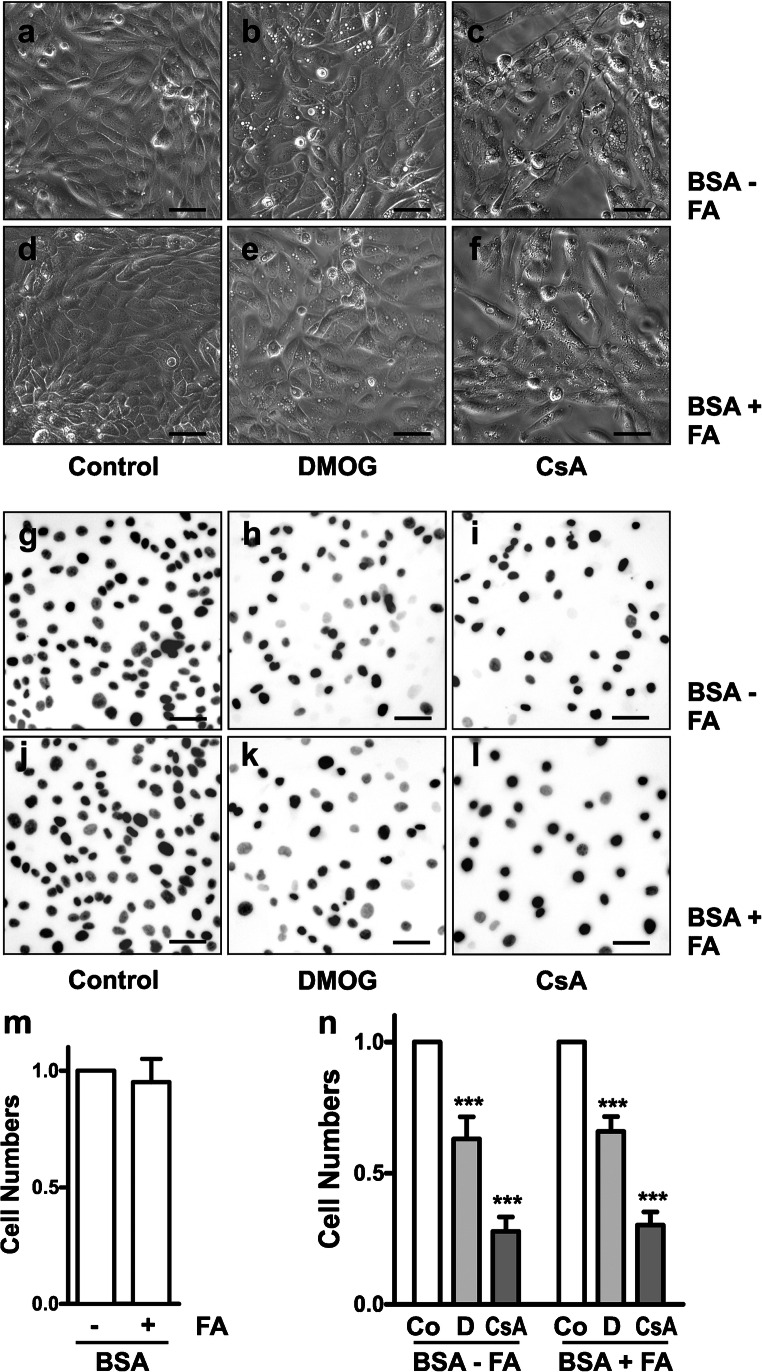
Lipid-loaded BSA does not induce cytotoxicity. hPTEC were incubated for 48 h in medium supplemented with 0.5% BSA essentially fatty acid-free (BSA-FA) or fatty acid-containing BSA (BSA + FA) as indicated. **a**–**f** Cells were treated with DMOG (1 mM) or CsA (10 μM) and imaged by phase-contrast microscopy. Scale bars, 50 μm. **g**–**l** Cells were treated as described above. Nuclei were stained with DAPI. Fluorescence microscopy images were converted to gray scale for better visualization. Scale bars, 50 μm. **m** Cell numbers were quantified by image analysis in hPTEC incubated with 0.5% BSA with or without FA. Cell numbers counted in FA-free BSA were set to 1 in each experiment. Control values were 1.00 ± 0.14, means ± SD, 6 isolations with 24 individual measurements. Data are means ± SEM of 6 isolations. **n** Cell numbers were quantified by image analysis in hPTEC incubated with DMOG (D, 1 mM) or CsA (10 μM). Cell numbers of control (Co) cells were set to 1 in each experimental setting. Control values were 1.00 ± 0.14 and 1.00 ± 0.13, respectively, means ± SD, 5 isolations with 20 individual measurements. Data are means ± SEM of 3–5 isolations. *** *p* < 0.001 compared to control cells

Human primary tubular epithelial cells (hPTEC) were isolated from healthy parts of human tumor nephrectomies. hPTEC showed typical morphological features (Fig. 2a, d): epithelial cells with cobble stone-like pattern, identified earlier as hPTEC of distal tubular origin, were surrounded by less adherent and more densely packed hPTEC of proximal tubular origin (Keller et al. 2012). These cells differ by their expression of cell-cell adhesion molecules: in human kidneys, proximal tubular cells express N-cadherin, whereas distal tubular cells express E-cadherin (Nouwen et al. 1993). In isolated tubular epithelial cells, the differential expression of cadherins is maintained, as we have shown earlier (Cicha et al. 2016; Keller et al. 2012). Based on their differential adhesion to plastic dishes, subcultures of more adherent distal and less adherent proximal hPTEC were obtained (Grampp and Goppelt-Struebe 2018) and analyzed for the mRNA expression of 12 markers specific for proximal or distal tubular cells (Lake et al. 2019; Lee et al. 2015) (Electronic Supplementary Material, Fig. S1a–n). N- and E-cadherin expression was verified on the mRNA level in proximal and distal hPTEC subcultures, respectively (Electronic Supplementary Material, Fig. S1a, d). Furthermore, distal hPTEC strongly expressed uromodulin (*UMOD*), cadherin 16 (*CDH16*) and aquaporin 2 (*AQP2*) as well as the glycolytic enzymes *HK1*, *PFKL*, *PKM* and *PFKM* (Electronic Supplementary Material, Fig. S1b, e, g, h, k, l, n). Subcultures enriched for proximal hPTEC showed high expression of *MIOX, AQP1, ALDOB, ASS1* and *GPX3* (Electronic Supplementary Material, Fig. S1c, f, i, j, m). These data confirmed E-cadherin and N-cadherin as reliable markers of distal and proximal hPTEC respectively.

hPTEC were incubated with 0.5% BSA as a source of naturally occurring lipids. To eliminate specific effects of BSA as a lipid carrier, cells were incubated with either FA-bearing (BSA + FA) or FA-depleted BSA (BSA-FA). Whereas high concentrations (3%) of lipid-loaded BSA (Arici et al. 2003) may have cytotoxic effects, in our experiments with 0.5% FA-loaded BSA, cell morphology (Fig. 2a, d) or cell numbers (Fig. 2g–m) did not differ from hPTEC cultured with FA-free BSA.

The effects of the PHD inhibitor DMOG were analyzed in comparison to the calcineurin inhibitor cyclosporine A (CsA), since proximal tubular lipid accumulation is a typical histopathological sign of CsA nephrotoxicity (Lhotak et al. 2012; Mihatsch et al. 1988). As both DMOG (Schultz et al. 2009) and CsA (Healy et al. 1998) may have antiproliferative and/or cytotoxic effects, we first analyzed DMOG- or CsA-treated hPTEC for alterations of cell morphology and cell numbers. Irrespective of the lipid content of BSA, DMOG (1 mM) and CsA (10 μM) reduced hPTEC numbers during 48 h (Fig. 2g–l). Reduction of cell numbers to 60% (DMOG) and 30% (CsA), respectively, was consistently observed in cell preparations obtained from different donors (Fig. 2n). Phase-contrast microscopy revealed significant morphological differences between DMOG- and CsA-treated hPTEC: DMOG-treated cells formed intact monolayers with intercellular contacts (Fig. 2b, e), suggesting that DMOG inhibited cell proliferation (Hubbi and Semenza 2015). By contrast, CsA-treated cells displayed a more elongated shape with long extensions and lost cell-cell contacts (Fig. 2c, f), indicating cell toxicity.

### DMOG increases lipid accumulation

**Fig. 3:**
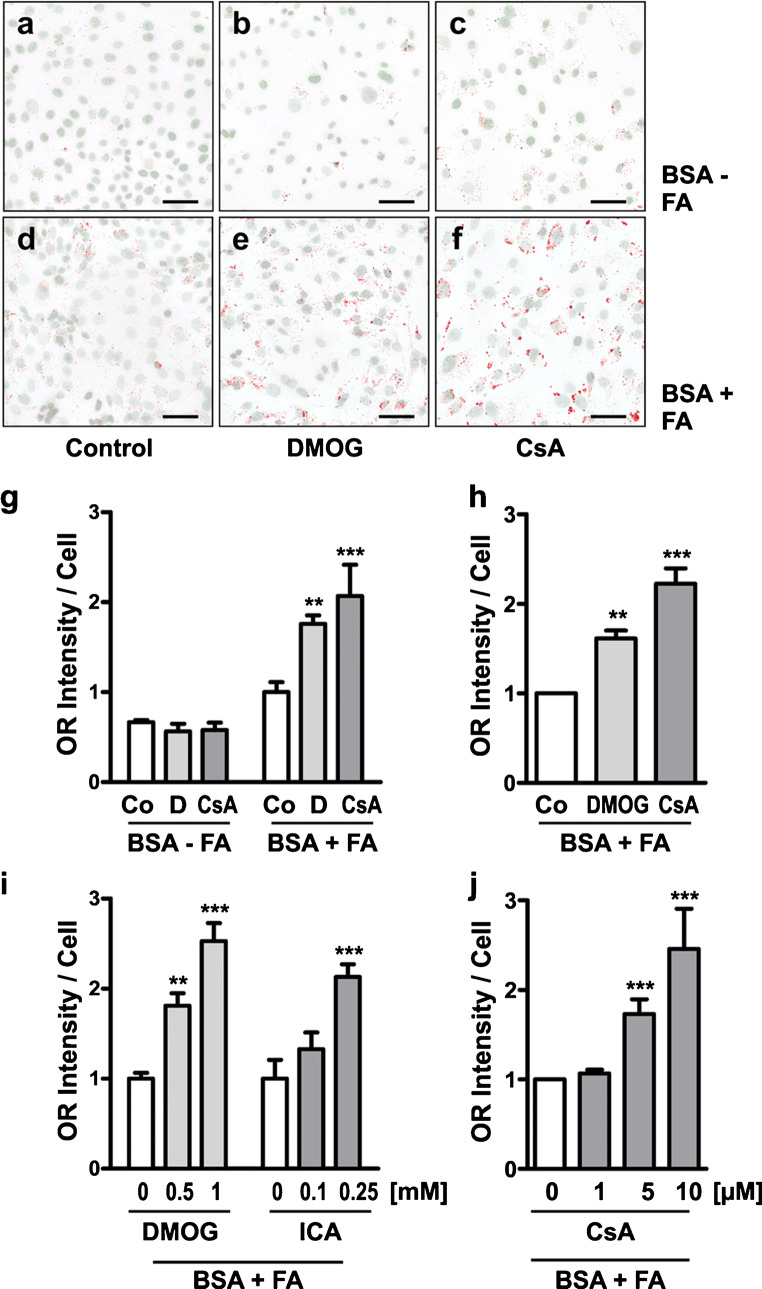
DMOG and CsA increase intracellular lipid accumulation from extracellular sources. hPTEC were incubated for 48 h in medium supplemented with 0.5% BSA essentially fatty acid-free (BSA-FA) or fatty acid-containing BSA (BSA + FA) as indicated. Oil Red staining was analyzed by microscopy (**a**–**f**) and by an infrared imaging system (**g**–**j**). **a**–**f** hPTEC were stimulated with DMOG (1 mM) or CsA (10 μM). Lipid droplets were stained with Oil Red and nuclei were visualized with DAPI. Scale bars, 60 μm. **g** hPTEC were incubated with vehicle control (Co), DMOG (1 mM) or CsA (10 μM) as indicated. OR staining intensity was determined in 48-well plates using the LI-COR Odyssey system and related to cell numbers. Data are means ± SD of quadruplicate wells of one representative experiment **p < 0.01, ***p < 0.001 compared to control cells. **h** hPTEC preparations obtained from 10 different donors were incubated with DMOG (1 mM) or CsA (10 μM). OR staining intensity was related to cell numbers. In each experiment mean intensity determined in control cells was set to 1. Control values were 1.00 ± 0.10, means ± SD, 10 isolations with 40 individual measurements. Data are means ± SEM. **p < 0.01, ***p < 0.001 compared to control cells. **i** hPTEC were incubated with the PHD inhibitors DMOG or ICA at the concentrations indicated. Data are means ± SD of one representative experiment analyzed in quadruplicate wells. Mean values of control cells were set to 1 in each condition. **p < 0.01, ***p < 0.001 compared to control cells. **j** hPTEC were incubated with increasing concentrations of CsA. Data are means ± SEM of 3 independent experiments each performed in quadruplicate wells. *** *p* < 0.001 compared to control cells. Control values were 1.00 ± 0.13, means ± SD, 3 isolations with 12 individual measurements

We then analyzed the effects of DMOG on lipid accumulation in hPTEC. CsA served as positive control. When FA were not available in the cell culture medium (Fig. 3a–c), hPTEC contained only very few and small lipid droplets under control conditions and under treatment with DMOG or CsA. In the presence of FA, DMOG and CsA apparently increased the amount and size of lipid droplets per cell (Fig. 3d–f).

For further quantitative analysis of lipid accumulation in hPTEC, we measured OR staining intensity per cell using a photometric assay. As suggested by the microscopy studies in Fig. 3(a–f), photometric quantification of OR staining verified that lipid accumulation in hPTEC depended on exogenous FA availability (Fig. 3g). Hardly any lipid accumulation was detected when hPTEC were incubated with FA-free BSA. In the presence of FA-loaded BSA, OR intensity increased in vehicle-treated cells and even more prominently in hPTEC treated with DMOG or CsA (Fig. 3g). These results were confirmed in 10 independent hPTEC preparations cultured with FA-bearing BSA (Fig. 3h). The PHD inhibitor ICA also augmented OR intensity in hPTEC corroborating the effects of DMOG (Fig. 3i). Lipid accumulation induced by CsA and both PHD inhibitors, DMOG and ICA, was concentration-dependent (Fig. 3i, j).

Our experiments so far demonstrated that 0.5% FA-loaded BSA did not provoke lipotoxicity in hPTEC but it was required for lipid accumulation induced by two different PHD inhibitors and CsA. Therefore, all the following experiments were carried out with FA-loaded BSA.

### Lipid accumulation in proximal and distal tubular epithelial cells

**Fig. 4:**
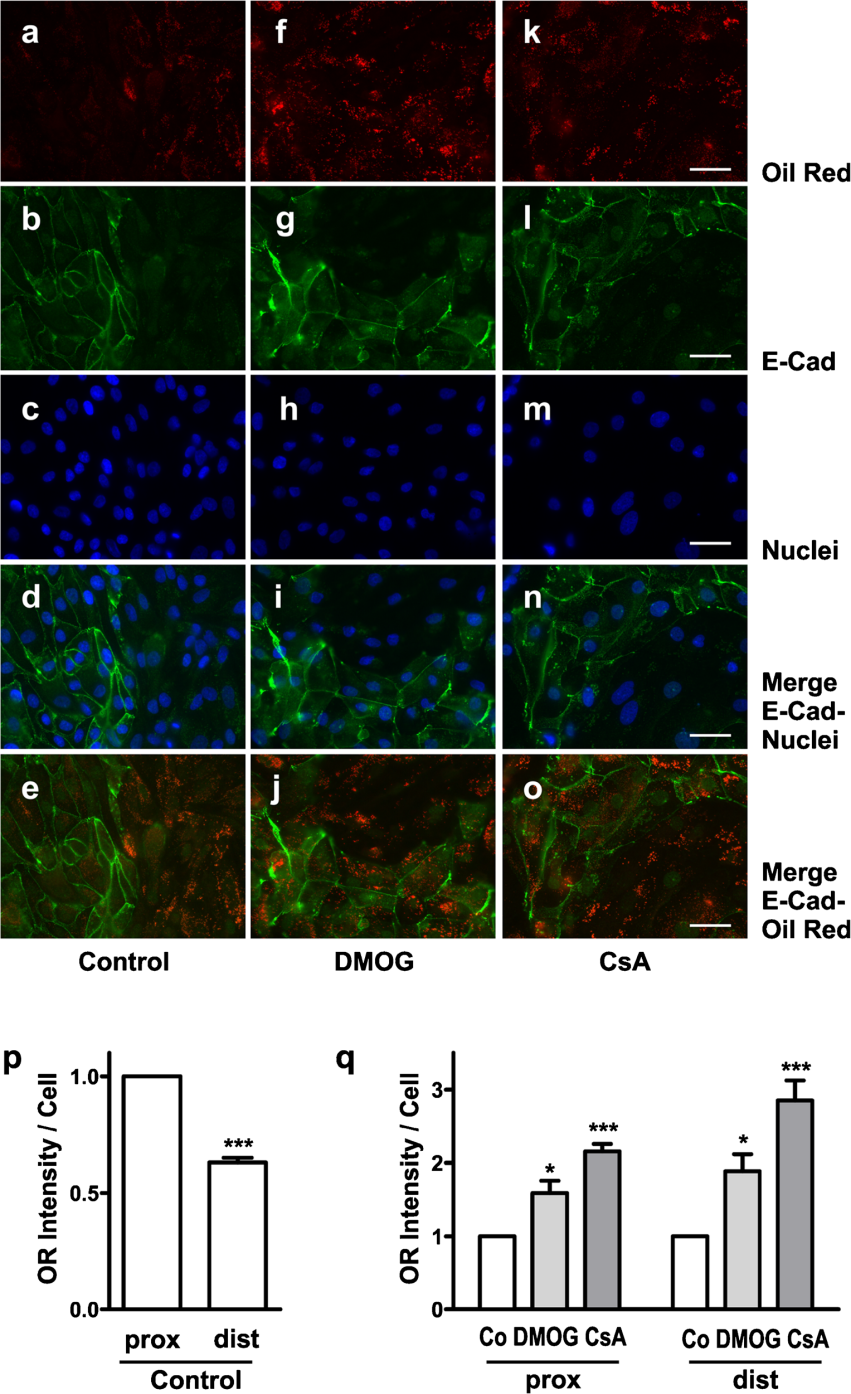
Lipid accumulation in proximal and distal tubular epithelial cells. **a**–**o** hPTEC were incubated with vehicle control, DMOG (1 mM) or CsA (10 μM) for 48 h in the presence of exogenous FA. Lipid droplets (Oil Red), E-cadherin and nuclei (DAPI) were detected by immunofluorescence microscopy. Scale bars, 40 μm. **p** OR staining intensity and numbers of nuclei were quantified in 3–6 areas of proximal and distal hPTEC on the same slide. The ratio of distal to proximal intensity per cell was determined for each slide. Control values were 1.00 ± 0.17 in proximal and 1.00 ± 0.19 in distal cells, 6 experiments with 23 individual measurements. Data are means ± SEM of 6 independent experiments. ***p < 0.001. **q** hPTEC were incubated with DMOG (1 mM) or CsA (10 μM) for 48 h. OR staining intensity per cells was determined as described in **p**. Mean values of control cells were set to 1 in each experiment. Data are means ± SEM of 5–7 independent experiments. * *p* < 0.05, ***p < 0.001 compared to control cells

To analyze if proximal and distal tubular cells differ in their capacity to utilize exogenous FA, lipid accumulation was specifically assessed in proximal and distal hPTEC by microscopical evaluation. OR staining intensity was quantified separately in areas of E-cadherin positive or negative confluent cells, representing distal and proximal hPTEC, respectively and related to cells numbers (Fig. 4a–e). Under control conditions, OR-stained lipid droplets were detected in both proximal and distal hPTEC but as expected predominantly in E-cadherin negative proximal hPTEC (Fig. 4a–e, quantification in Fig. 4p). Stimulation with either CsA or DMOG increased lipid accumulation in proximal and distal cells (Fig. 4f–o, quantification in Fig. 4q). Due to the lower baseline (Fig. 4p), the increase appeared even more prominent in distal cells compared to proximal cells (Fig. 4q). These data indicate that pharmacologic modulation of lipid accumulation is not restricted to proximal tubular cells. Evidently, also distal tubular cells have the capacity to handle exogenous FA.

### DMOG and CsA differentially upregulate *PLIN2* and *PLIN4*

**Fig. 5:**
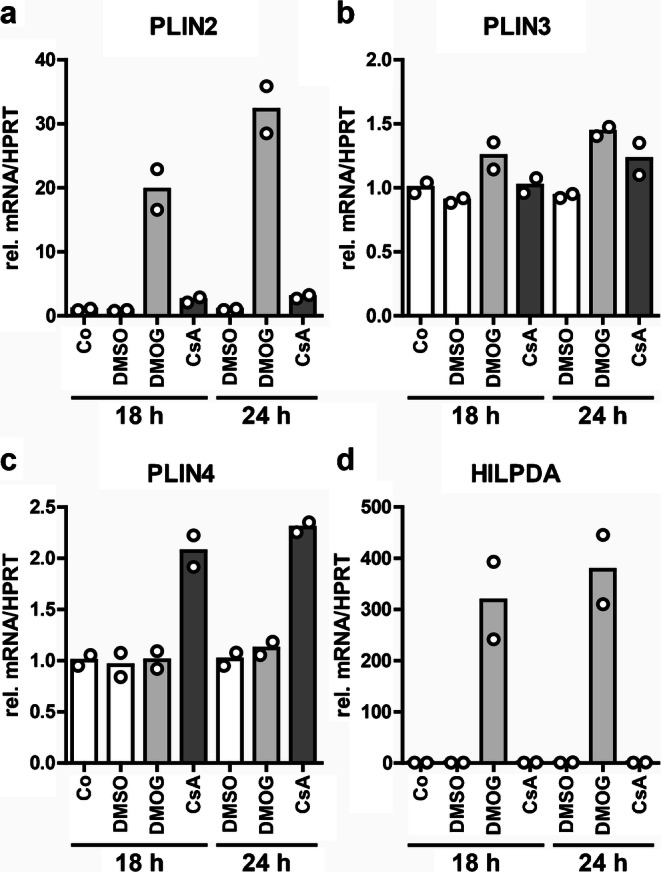
DMOG and CsA differentially regulate perilipins. **a**–**d** hPTEC were incubated with medium control (Co), vehicle control (DMSO), DMOG (1 mM) or CsA (10 μM) for 18 h or 24 h and RNA was isolated from cell lysates. mRNA expression of *PLIN2*, *PLIN3*, *PLIN4* and *HILPDA* was determined by real-time PCR. Data are individual values and means of 2 isolations

As PHDi might alter the composition of lipid droplets, we analyzed the mRNA expression of perilipins, which are some of the most abundant lipid droplet proteins (Sztalryd and Brasaemle 2017). *PLIN2*, *PLIN3* and *PLIN4* were robustly expressed in hPTEC, whereas *PLIN1* and *PLIN5* were only slightly detectable. DMOG increased the mRNA expression of *PLIN2* as well as the hypoxia-inducible lipid droplet-associated (*HILPDA*) gene (Fig. 5a, d), whereas CsA selectively upregulated *PLIN4* mRNA (Fig. 5c). *PLIN3* was not regulated by either CsA or DMOG (Fig. 5b).

### Role of ER stress in lipid accumulation

**Fig. 6:**
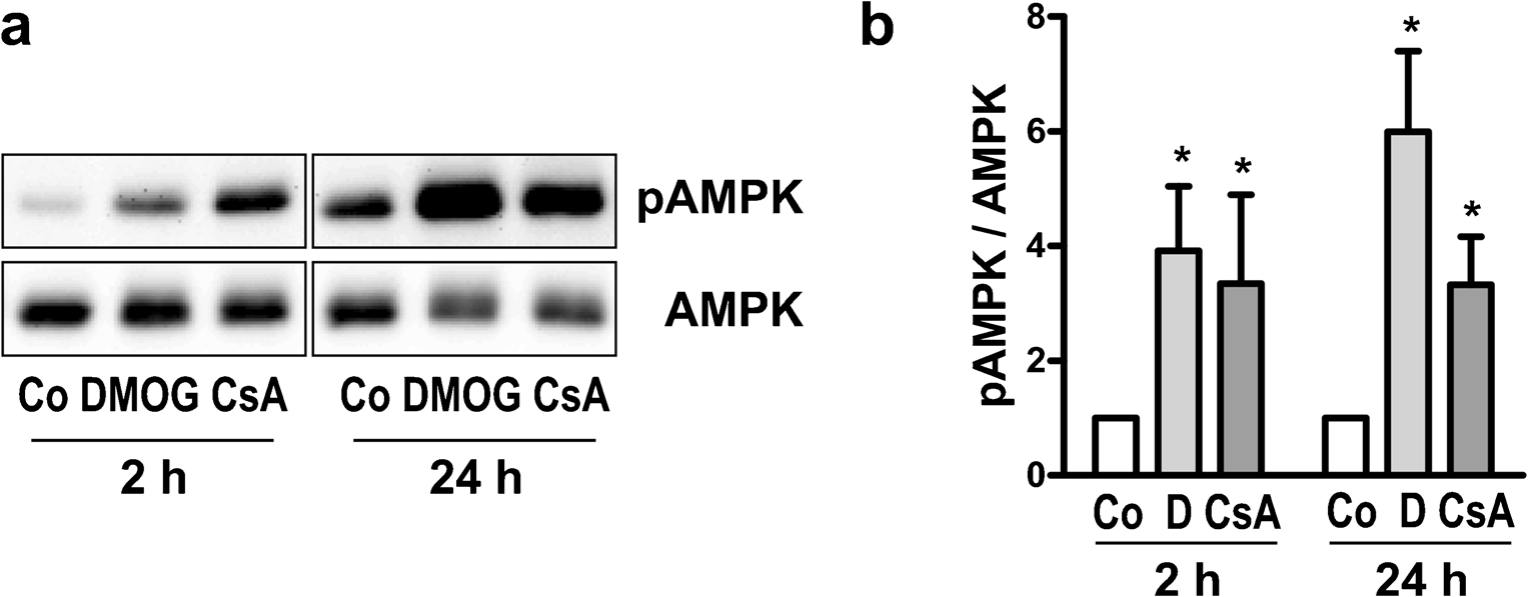
DMOG and CsA activate AMPK. **a** hPTEC were incubated with DMOG (1 mM) or CsA (10 μM) for 2 h or 24 h. Expression of pAMPK and AMPK was detected by Western blotting. **b** Summary of densitometric quantification obtained with cells isolated from 3 different donors. Ratio of pAMPK/AMPK at 2 h and 24 h was set to 1 for each blot. *p < 0.05 compared to control cells

**Fig. 7:**
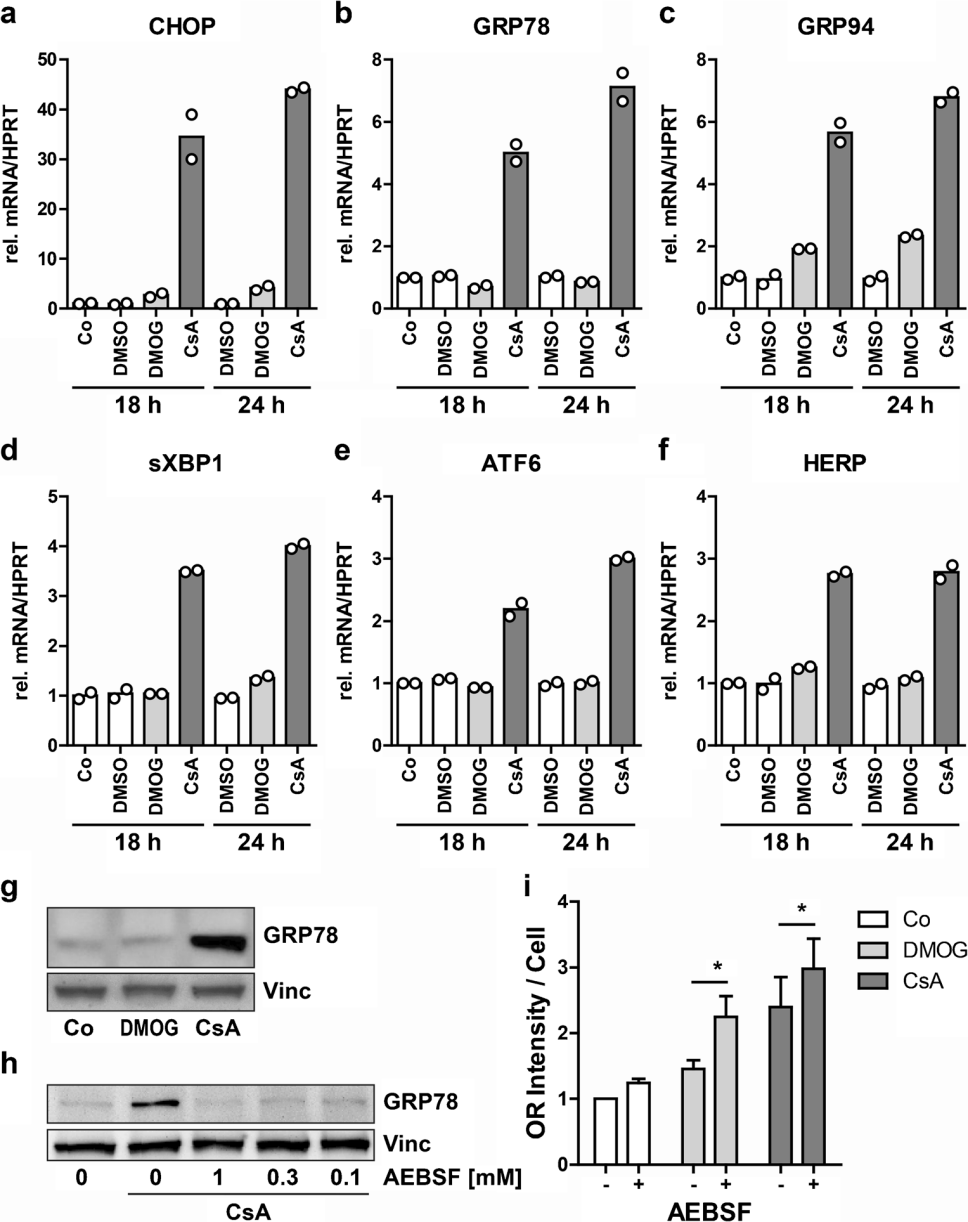
DMOG does not activate ER stress in hPTEC. **a**–**f** RNA was isolated from hPTEC incubated with medium control (Co), vehicle control (DMSO), DMOG (1 mM) or CsA (10 μM) for 18 h or 24 h. mRNA expression of markers for ER stress (*CHOP, GRP78, GRP94, sXBP1, ATF6* and *HERP*) was determined by real-time PCR. Data are individual values and means of 2 isolations. **g** Expression of GRP78 was detected by Western blotting in hPTEC incubated for 24 h with DMOG (1 mM) or CsA (10 μM). Vinculin (Vinc) was used as loading control. **h** Western blot analysis of GRP78 expression in hPTEC incubated with CsA (10 μm) and the protease inhibitor AEBSF at the indicated concentrations for 24 h. Vinculin (Vinc) was used as loading control. **i** hPTEC were incubated for 24 h with DMOG (1 mM) or CsA (10 μM) and with or without AEBSF (0.1 mM) and stained with Oil Red (OR). OR staining intensity was quantified photometrically. Control values were 1.00 ± 0.15, means ± SD, 3 experiments with 12 individual measurements. Data are means ± SEM of 3 experiments. *p < 0.05 cells with AEBSF compared to cells without AEBSF

AMP-activated protein kinase (AMPK) is an energy sensor that has a key role in the regulation of protein and lipid metabolism (Rajani et al. 2017). Next, we assessed if AMPK activation was modified by PHD inhibition. Western blot analysis showed a time-dependent phosphorylation of AMPK, when hPTEC were treated with DMOG or CsA for 2 or 24 h (Fig. 6a, b). AMPK activation is supposed to reduce endoplasmic reticulum (ER) stress, which is typically induced by CsA in many cell types, among them tubular cell lines (Cheng et al. 2012; Lhotak et al. 2012; Pallet et al. 2008). Therefore, we analyzed the mRNA expression of several markers of ER stress, namely *ATF6*, *CHOP*, *GRP78*, *GRP94*, *HERP* and *sXBP1* (Bouvier et al. 2009, 2012; Fougeray et al. 2011) (Fig. 7a–f). All ER stress markers were significantly upregulated by CsA, whereas DMOG did not markedly alter the expression of these markers. CsA-mediated upregulation of GRP78, one of the most prominent markers for ER stress (Kim et al. 2008), was also confirmed at the protein level (Fig. 7g). In line with the mRNA data, GRP78 protein was not upregulated by DMOG. So far, our experiments showed that both DMOG and CsA increased lipid accumulation and activated AMPK in hPTEC but they differentially modulated the cellular stress response.

To analyze the role of ER stress in lipid accumulation, we employed the protease inhibitor AEBSF, which prevented CsA-mediated upregulation of GRP78 and lipid accumulation in HK-2 cells (Lhotak et al. 2012). In hPTEC, AEBSF completely prevented the CsA-induced upregulation of GRP78 at a concentration of 0.1 mM (Fig. 7h) but significantly increased lipid accumulation provoked by CsA and also by DMOG (Fig. 7i) implying that CsA- and DMOG-induced lipid accumulation was independent of ER stress in hPTEC.

## Discussion

Pharmacological or genetic inactivation of the oxygen-sensing PHDs leads to HIFα stabilization and activation of the HIF signaling pathways (Schodel and Ratcliffe 2019). PHD2 is the most important of the three PHD isoforms in the hypoxic response (Berra et al. 2003), the most abundant isoform in rodent kidneys (Schodel et al. 2009) and indispensable in mice (Takeda et al. 2006). In this study, targeted deletion of tubular *Phd2* enhanced lipid droplet accumulation in proximal tubular cells in vivo. In hPTEC, PHD inhibition augmented lipid accumulation in proximal as well as distal tubular cells in the presence of extracellular FA. In contrast to CsA, inhibition of PHDs did not induce cell injury and ER stress.

The role of the HIF pathway in the regulation of renal lipid metabolism has been intensively examined in ccRCC (Mylonis et al. 2019). However, beyond tumor models, the effects of HIF on lipid metabolism in the kidney are largely unknown. Recently, lipid accumulation was observed in proximal tubules of embryonic *vhl*-deficient zebrafish larvae (van Rooijen et al. 2018). In our in vivo experiments, we found larger lipid deposits in proximal renal tubules of *Phd2*^*ΔKsp*^ mice than in wild-type littermates. Correspondingly, pharmacological PHD inhibition stimulated lipid accumulation in isolated hPTEC, preferentially in proximal but also in distal tubular cells. Although FA and endogenous lipids are the preferred energy source of proximal tubular cells (Silva 1990), all nephron segments can take up FA and degrade them by β-oxidation in their mitochondria (Guder et al. 1986). Cortical proximal and distal nephron segments have a similar capacity of mitochondrial β-oxidation (Guder and Ross 1984). Moreover, esterification of FA to triacylglycerols also occurs in all nephron segments; however, its activity is higher in proximal than in distal tubular cells (Guder and Ross 1984). Thus, triacylglycerol deposits accumulate primarily in proximal tubular cells under conditions of increased FA supply but they were also occasionally found in the distal nephron (Wirthensohn and Guder 1986). Our observation clearly supports the notion that distal epithelial cells are also able to handle FA.

We chose serum albumin as a source of extracellular lipids because it binds the vast majority of FA in vivo (Ruan et al. 2009). Thus, lipid composition was not refined to specific FA but rather mimicked the in vivo situation in proteinuric patients and experimental animals. Exposure of proximal tubular cells in vitro (at concentrations > 3%) and in vivo to FA-loaded albumin causes cytotoxicity and aggravates tubulointerstitial injury (Arici et al. 2003; Kamijo et al. 2002; Thomas et al. 2002; van Timmeren et al. 2005). We did not observe changes in cell numbers, necrotic, or apoptotic cells when hPTEC were incubated with 0.5% FA-loaded BSA. However, the low extracellular FA concentration was sufficient to increase the intracellular lipid content in hPTEC. This finding is in line with previous reports that FA oxidation in the kidney is normally saturated at near-physiological concentrations resulting in decreased FA oxidation and increased lipid storage when FA supply rises (Wirthensohn and Guder 1986).

Lipid accumulation in droplets has been commonly found in multiple cell types under hypoxia and is attributed to decreased mitochondrial lipid catabolism and elevated lipid import, synthesis and storage (Thomas and Ashcroft 2019). Several proteins involved in these processes are directly regulated by HIFs, among them the lipid droplet-associated proteins PLIN2 and HILPDA. The HIF-dependent upregulation of *PLIN2* or *HILPDA* promoted lipid droplet formation and neutral lipid accumulation (Bensaad et al. 2014; Bildirici et al. 2018; Gimm et al. 2010; Qiu et al. 2015). These findings are consistent with our observations in hPTEC and might, at least partially, explain PDH-induced lipid accumulation in hPTEC.

Besides *PLIN2*, *PLIN3* and *PLIN4* were also expressed in hPTEC but they were not regulated by PHDi. *PLIN1* and *PLIN5* were barely detectable in hPTEC. In accordance with our data, PLIN2 and 3 have been ubiquitously found in mammalian cells and tissues, whereas the expression of PLIN1, 4 and 5 is spatially more restricted (Sztalryd and Brasaemle 2017). PLIN2 (adipophilin), PLIN3 (TIP47) and PLIN4 (S3-12) identified exogenously derived lipid droplets in human adipocytes (Heid et al. 2014). Accordingly, expression of *PLIN*2, *PLIN3* and *PLIN4* in hPTEC might indicate an exogenous source of lipid droplets.

PHDi-induced lipid accumulation in hPTEC was dependent on exogenous FA availability, which might imply that PHDi promoted lipid uptake. The import of extracellular FA into the cell is known to be increased by HIF-dependent upregulation of fatty acid binding proteins (FABP) 3, 4 and 7 (Mylonis et al. 2019). Therefore, upregulation of FABP might also have a role in PDH-induced lipid accumulation in hPTEC, which should be investigated in further experiments.

Transgenic mice with selective deletion of *Vhl* or *Phd2/3* in cardiac myocytes developed lipid accumulation (Lei et al. 2008; Moslehi et al. 2010). This finding relied on HIF-1α-dependent activation of peroxisome proliferator-activated receptor (PPAR)γ and subsequent stimulation of FA uptake and glycerolipid biosynthesis (Krishnan et al. 2009) as well as reduced DNA binding activity of PPARα to its heterodimer partner retinoid X receptor (RXR) resulting in suppression of mitochondrial FA metabolism (Belanger et al. 2007). In the kidney, PPARα is predominantly found in proximal tubules and PPARγ in medullary collecting duct and interstitial cells (Yang et al. 1999). Therefore, proximal tubular lipid accumulation in *Phd2*^ΔKsp^ mice and PHDi-treated hPTEC might also involve the PPARα pathway.

Besides hypoxia and HIF stabilization, lipid accumulation can also be mediated by ER stress (Han and Kaufman 2016). Interestingly, hypoxia and ER stress are mutually connected (Maekawa and Inagi 2017). In hPTEC, PHDi did not upregulate typical markers of ER stress, among them GRP78. However, the hypoxic regulation of ER stress seems to be cell type-dependent. In the proximal tubular cell lines HK-2 and NRK52E, HIF stabilization by hypoxia and cobalt chloride, respectively, did not trigger ER stress (Bouvier et al. 2012; Hiramatsu et al. 2007), whereas DMOG or cobalt chloride increased GRP78 expression in endothelial cells SVEC4-10 and HUVEC (Natarajan et al. 2009; Ostergaard et al. 2009). At the molecular level, inhibition of PHD3 but not PHD2 stabilized the unfolded protein response genes ATF-4 in HeLa cells (Koditz et al. 2007). As PHD2 is the major PHD isoform in tubular cells (Schodel et al. 2009), this might, at least partially, explain why PHDi did not induce GRP78 and other ER stress markers in hPTEC.

Furthermore, PLIN2-mediated formation of lipid droplets promoted ER homeostasis and suppressed ER stress responses in ccRCC (Qiu et al. 2015). Therefore, PHDi-induced upregulation of *PLIN2* in hPTEC might also contribute to the absence of ER stress.

HIF is linked to the AMPK signaling pathway. Both of them facilitate adaptation to cellular stress by energy deficiency and/or oxygen deprivation and have overlapping signaling targets (Salminen et al. 2016). PHDi activated AMPK in hPTEC, as it has been observed in HK-2 cells before (Li et al. 2015). DMOG-induced AMPK activation might also have a role in inhibiting GRP78 and thus protect hPTEC from ER stress, as activation of AMPK by metformin suppressed albumin-induced ER stress in HK-2 cells (Lee et al. 2012).

We compared the effects of PHDi with the immunosuppressant cyclosporine A (CsA), because CsA stimulates renal tubular lipid accumulation (Mihatsch et al. 1988). In line with earlier studies in proximal tubular cell lines HK-2 and LLC-PK_1_ (Healy et al. 1998; Lhotak et al. 2012), treatment of hPTEC with CsA resulted in a concentration-dependent increase in lipid accumulation that was associated with alterations of cell morphology, increased cell death and expression of diverse markers of ER stress, among them GRP78. Activation of GRP78 was involved in the development of CsA-induced tubular cytotoxicity, tubular vacuolization, lipid accumulation and apoptotic cell death (Cheng et al. 2012; Lhotak et al. 2012). Enhanced expression of GRP78 was also found in kidney biopsies from CsA-treated patients (Lindenmeyer et al. 2008). Blocking GRP78 upregulation by the protease inhibitor AEBSF prevented CsA-induced lipid accumulation in HK-2 cells (Lhotak et al. 2012), while in hPTEC, AEBSF further increased lipid accumulation. This opposing effect might be cell type-specific and/or attributed to other metabolic activities of AEBSF, which was reported to inhibit sterol regulatory element-binding protein-2 (SREBP-2) (Lhotak et al. 2012), phospholipase D (Hirota et al. 2002), or NADPH oxidase (Vesey et al. 2005).

Just as DMOG, CsA activated AMPK in hPTECs, which has previously been reported in HK-2 cells as well (Yadav et al. 2015). The functional role of AMPK activation in the context of CsA toxicity in hPTEC needs further investigation and might involve metabolic effects of AMPK, which are not related to changes in lipid metabolism.

Furthermore, CsA selectively upregulated *PLIN4* in hPTEC. The effect of CsA on perilipins and other lipid droplet proteins is unclear and needs further investigation. Drug-induced accumulation of lipid droplets in human hepatoma cells was associated with upregulation of *PLIN4* (Antherieu et al. 2011). Therefore, lipid accumulation following CsA treatment might not only be a consequence of cellular toxicity producing fatty degeneration but might also involve enhanced expression of lipogenic genes.

Triacylglycerol formation and storage in lipid droplets may, beyond functioning as metabolic energy depots (Welte and Gould 2017), represent a cellular cytoprotective response (Listenberger et al. 2003). It is tempting to speculate that increased renal tubular deposition of neutral lipids due to pharmacological inhibition of PHDs contributes to the renoprotective effects of PHDi against experimental of AKI and CKD (Haase 2017; Nangaku et al. 2013). PHDi are presently evaluated in clinical trials for the treatment of renal anemia in CKD (Sugahara et al. 2017). Patients with CKD may thus benefit from PHDi not only as treatment for renal anemia but also as regulators of lipid metabolism in renal tubular cells.

## Electronic supplementary material

Fig S1 (PDF 740 kb)

Table S1 (PDF 45 kb)
