# Supplementary material for: Inhibition of oxygen-sensing prolyl hydroxylases increases lipid accumulation in human primary tubular epithelial cells without inducing ER stress
Source: Cell Tissue Res. 2020 Mar 18;381(1):125–40. doi: 10.1007/s00441-020-03186-w (PMC7306052; doi:10.1007/s00441-020-03186-w)
Supplement: Supplementary file 1 — (PDF 740 kb) [file 441_2020_3186_MOESM1_ESM.pdf]

Figure S1: Characterization of hPTEC preparations enriched for proximal and distal tubular cells
hPTEC isolated from 2 donors were separated into subcultures enriched for proximal and distal tubular cells. mRNA expression of tubular segment-specific markers *NCAD* (**a**), *UMOD* (**b**), *MIOX* (**c**), *ECAD* (**d**), *CDH16* (**e**), *AQP1* (**f**), *AQP2* (**g**), *HK1* (**h**), *ALDOB* (**i**), *ASS1* (**j**), *PFKL* (**k**), *PKM* (**l**), *GPX3* (**m**) and *PFKL* (**n**) were detected by real-time PCR.

Figure S1

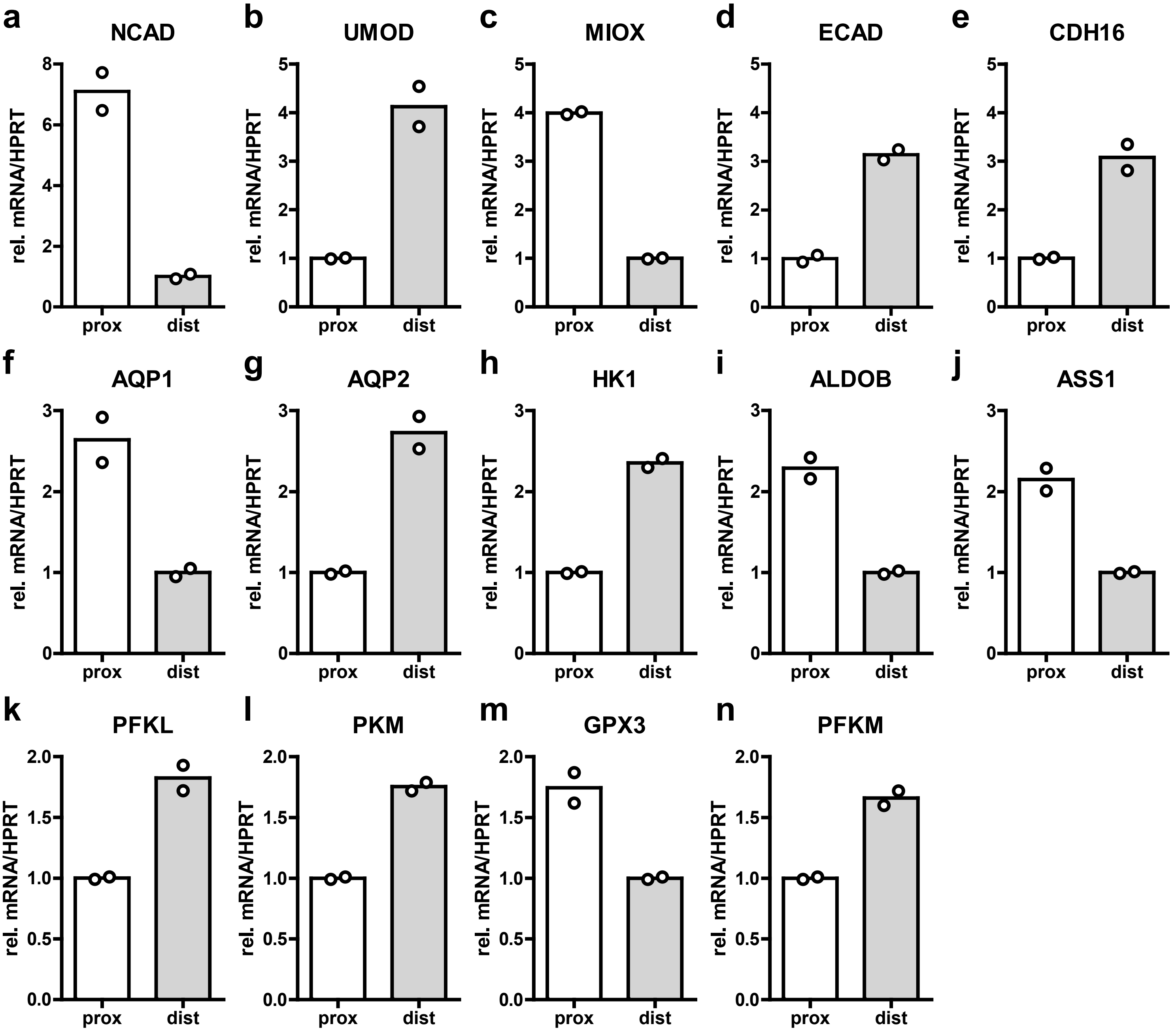
a
NCAD
b
UMOD
c
MIOX
d
ECAD
e
CDH16
f
AQP1
g
AQP2
h
HK1
i
ALDOB
j
ASS1
k
PFKL
l
PKM
m
GPX3
n
PFKM
rel. mRNA/HPRT
prox
dist
