# Supplementary material for: Inhibition of oxygen-sensing prolyl hydroxylases increases lipid accumulation in human primary tubular epithelial cells without inducing ER stress
Source: Cell Tissue Res. 2020 Mar 18;381(1):125–40. doi: 10.1007/s00441-020-03186-w (PMC7306052; doi:10.1007/s00441-020-03186-w)
Supplement: Supplementary file 2 — (PDF 45 kb) [file 441_2020_3186_MOESM2_ESM.pdf]

Table S1: Human primer sequences used for quantitative real-time PCR analysis

Gene name	NCBI RefSeq access number	Primer sequence (5‘ to 3‘)
ALDOB	NM_000035.4	Fwd: AAAGCCATTCACAATACGAAGCC<br>Rev: GACAAGGTAGTCCTGTTTCACCA
AQP1	NM_198098.3	Fwd: TTGGACACCTCCTGGCTATTGA<br>Rev: TCCAGTGGTTGCTGAAGTTGTG
AQP2	NM_000486.5	Fwd: CAGGCAATACCCATCCATCACC<br>Rev: GGAGAGGCAGACAGAGAAGGAA
ASS1	NM_000050.4	Fwd: CCCAGATAAAGGTCATTGCTCCC<br>Rev: TTGCGTACTCCATCAGGTCATTG
ATF6	NM_007348.4	Fwd: CCAGCAGCACCCAAGACTCAAACA<br>Rev: GTGTGACTCCCCCAGCAACAGC
CDH16	NM_004062.4	Fwd: GTCCCTAGAGCCTATCCACCTG<br>Rev: TGCATTCACTTCAAAGGGTCCC
CHOP	NM_001195053.1	Fwd: TGGAAGCCTGGTATGAGGAC<br>Rev: TGTGACCTCTGCTGGTTCTG
ECAD	NM_004360.5	Fwd: CATTGCCACATACACTCTCTTCTCTC<br>Rev: TGCATTCCCGTTGGATGAC
GPX3	NM_002084.5	Fwd: CACTACAGGAAGAGCTTGCACC<br>Rev: CTGGTCGGACATACTTGAGGGT
GRP78	NM_005347.5	Fwd: GGTGAAAGACCCCTGACAAA<br>Rev: GTCAGGCGATTCTGGTCATT
GRP94	NM_003299.3	Fwd: AGTACGGATGGTCTGGCAAC<br>Rev: TGAGGCGAAGCATTCTTTCT
HERP	NM_014685.4	Fwd: CATCAGGGGCTTTTGTTCC<br>Rev: GATTGGCAGGCTGGTTTTC
HILPDA	NM_013332.4	Fwd: TGGAGTGTTCAGTGCCCATTT<br>Rev: CCACCCCCTTCAGACCAATAC
HK1	NM_000188.2	Fwd: CAGATAGAGGAGACCCTGGCTC<br>Rev: CTTAACCACGGCATTGTTGTGC
HPRT	NM_000194.3	Fwd: GACCAGTCAACAGGGGACA<br>Rev: AACACTTCGTGGGGTCCTTTTC
MIOX	NM_017584.6	Fwd: GCTTTCTACATGATCCGGTTCCAC<br>Rev: TTGGTGTAGAGGTCGAACTTGTTG
NCAD	NM_001792.4	Fwd: CCCTGCTTCAGGCGTCTGTA<br>Rev: TGCTTGCATAATGCGATTTCACC
PFKL	NM_001002021.3	Fwd: TGTGTAACCAGGGTAGAGGTCG<br>Rev: AAAGAAACAGTCTGTCCCAGCG

PFKM	NM_001166686.2	Fwd: CCCATTTGTGGTCATTCCTGCT<br>Rev: ATGATAAACACCCGACGCTTGG
PKM	NM_001206796.3	Fwd: GTGTGACGAGAACATCCTGTGG<br>Rev: CACCTTTCTGCTTCACCTGGAG
PLIN1	NM_002666.5	Fwd: TCACAGCCACATTTCCATTTG<br>Rev: TCACCATTTTGGTTCCCCAG
PLIN2	NM_001122.4	Fwd: TGCTGTGACGACTACTGTGAC<br>Rev: TTAATGCTGCCACTGACCACA
PLIN3	NM_005817.5	Fwd: GCCCAGAACACACCTGTCAC<br>Rev: GGGTTGAGGACTCCAGAGCA
PLIN4	NM_001367868.2	Fwd: ATGCTGAAAGTGGGTCTCCG<br>Rev: GCCTGCCTTGGGATGATGAT
PLIN5	NM_001013706.3	Fwd: ATCACTTCCTGCCCATGACG<br>Rev: CTCCACCGAACCCACTTCAG
UMOD	NM_003361.3	Fwd: CTCGGGCTTCAATGACAGAGAC<br>Rev: GCATGGGTTTCATTCCTCGTCA
sXBP1	NM_001079539.1	Fwd: GCAGGTGCAGGCCCAGTTGT<br>Rev: TGGGTCCAAGTTGTCCAGAATGC
